# Comparison of some meat quality and liver characteristics in Muscovy and mule ducks

**DOI:** 10.5194/aab-63-137-2020

**Published:** 2020-05-05

**Authors:** Dariusz Kokoszyński, Anna Wilkanowska, Henrieta Arpášová, Cyril Hrnčár

**Affiliations:** 1 Department of Animal Sciences, Faculty of Animal Science and Biology, UTP University of Science and Technology, Bydgoszcz, 85084, Poland; 2 Department of Small Animal Science, Faculty of Agrobiology and Food Resources, Slovak University of Agriculture, Nitra, 94976, Slovakia

## Abstract

The aim of the study was to compare Muscovy ducks and mule ducks for proximate analysis, colour attributes, sensory properties of the meat, and concentration of some minerals in the meat and liver, with consideration of the effect of sex on the analysed traits. The study used 46 Muscovy and 44 mule ducks. At the end of the rearing period, 40 birds (10 males and 10 females of each genotype) were selected for slaughter. Meat and liver samples were collected from the slaughtered birds to determine quality traits. The analysed ducks of different genotypes differed significantly in the water content, sodium content, and redness of breast muscles; in the water, fat, and zinc content of leg muscles; and in the sodium, iron, and copper content of liver. Regardless of genotype, males had a higher sodium content in breast muscles; contained more protein, sodium, phosphorus, and magnesium and less potassium and copper in leg muscles; and had a significantly higher content of sodium, potassium, phosphorus, magnesium, zinc, iron, and copper in liver compared to females. The genotype–sex interaction was significant for the sodium and potassium content of breast muscles, for the water, protein, sodium, potassium, phosphorus, magnesium, and copper content of leg muscles, and for the copper content of liver. Our study provided information about differences in the quality of meat and liver in Muscovy and mule ducks.

## 1 Introduction

In recent years, there has been a rapid increase in the world production of duck meat. In 2000–2017 (FAOSTAT, 2019) duck meat production increased by 53.8 % (from $2.90\times 10^{6}$ to $4.46\times 10^{6}$ t), which is more than the production of goose meat (34.0 %, from $1.88\times 10^{6}$ to $2.52\times 10^{6}$ t) and turkey meat (15.8 %, from $5.13\times 10^{6}$ to $5.94\times 10^{6}$ t) and less than the production of chicken meat (86.0 %, from $58.7\times 10^{6}$ to $109.0\times 10^{6}$ t). Despite such a high increase, in 2017 duck meat accounted for only 3.7 % of the total poultry meat production. Production of fattening ducks is limited by a lack of tradition of consuming duck meat and its preparations in most countries of the world, the consumer belief that duck carcasses are high in fat and energy, the red colour of the meat, and the higher price of carcasses, carcass components, and meat preparations from ducks compared to broiler chickens.

The main producer of duck meat in the world is Asia (especially China), which supplies around 84 % of the global duck meat production. In Asia, the principal breed of ducks used for meat production is Pekin. In Europe, which supplies around 11 % of world duck meat production, Pekin dominates in most countries (especially in northern and central eastern Europe), while mule and Muscovy ducks predominate in the Mediterranean countries (especially in France and Italy) (Retailleau, 1999; Wołoszyn, 2002; Mazurowski et al., 2016)

Muscovy ducks owe their popularity not only to the conformation traits but also to the slightly higher content of breast and leg meat, and a lower content of skin with subcutaneous fat and abdominal fat in the carcass, which better meets the requirements of the consumers of duck meat compared to Pekin duck carcasses. Under the intensive system, female Muscovy ducks are raised to 9–10 weeks, and males are raised to 11–12 weeks of age. Despite the many advantages, Muscovy duck production is not significant in many countries of the world, due to the longer rearing period, higher prophylaxis and nutrition costs, lower egg production, and worse conversion of feed to kilograms of meat or eggs compared to Pekin ducks (Bednarczyk, 1981; Biesiada-Drzazga, 2012; Różewicz and Kaszperuk, 2017).

Mule ducks are relatively tolerant of adverse environmental conditions, temperature changes, and diseases. They exhibit a relatively fast rate of growth, rather good feed conversion, and good meat quality. Intensively raised broiler mule ducks are slaughtered at 11–12 weeks of age or fattened to produce foie gras. Production of mule ducks is a specialty of France, where they are used for foie gras production. Only male mule ducks are force-fattened in France (females have lower liver weight), compared to ducks of both sexes in Taiwan (Wołoszyn, 2002). In France, 97 % of foie gras comes from male mule ducks, and the meat of mule ducks accounts for around 58 % of the global duck meat production in this country (Braine, 2010).

In the last dozen years or so, relatively few research publications compared the quality of meat from Muscovy and mule ducks (Wawro et al., 2004; Chartrin et al., 2006; Lucia et al., 2008; Khaziev et al., 2018), which provided an incentive to undertake the present study.

The objective of the study was to compare the commercial hybrids of Muscovy and mule ducks for basic chemical composition, content of some minerals, physicochemical and sensory properties of the breast and/or leg meat, and the content of selected minerals in the liver. The obtained results allowed us to determine differences in the nutritive and technological value of Muscovy and mule duck meat and to point out which ducks better meet the requirements of duck meat processors and consumers.

## 2 Materials and methods

### 2.1 Birds and housing

The study used 46 Muscovy ducks (21 males and 25 females) derived from drake MMLCFFCZCK and duck CRAMMLCFF, with parents imported from the French company Grimaud Frères, and 44 mule ducks of the STE MULARD set (23 males and 21 females) derived from parents (male Muscovy duck and female Pekin duck) imported from the French company Couvoir de la Mésangère. Muscovy ducks and mule broilers were purchased in Poland. The 1 d old sexed ducklings were brought to the farm from a hatchery, wing-banded with padlock tags, and grouped by genotype and sex. Ducks were kept on deep litter in four pens (each with an area of 12 m${}^{2}$) in a closed facility without outdoor access. Incandescent light was used in the building and a light programme of 16 L : 8 D (light : dark hours) was used except for the first 3 d of life (24 L). In the first 21 d of rearing, infrared lamps were used as an additional source of heat. Temperature, relative humidity, and air movement were adjusted to bird age. The study was conducted with approval of the Local Ethics Committee on Vertebrate Experimentation in Bydgoszcz, Poland (resolution no. 27/2012).

### 2.2 Feeding programme and diets

Throughout rearing, ducks had 24 h access to water and were fed commercial diets ad libitum. From 1 to 21 d, ducks received a starter diet in crumble form, which contained 20.62 % crude protein (CP) and 12.19 MJ metabolizable energy (ME) per kilogram. From 22 to 70 d (Muscovy females) or from 22 to 84 d (Muscovy males and mule males and females) were fed a commercial grower/finisher diet containing 17.59 % CP and 12.68 MJ ME per kilogram. The data for ingredient composition and the basic chemical composition, energy value and content of selected amino acids, and calcium and available phosphorus of the starter and grower/finisher diets fed to ducks are shown in Table 1.

**Table 1:** Ingredients and chemical composition of the diets for ducks.

Ingredients	Starter	Grower/finisher
(g kg${}^{-1}$ of feed))		
	1–21 d	22–84 d
Corn	350.6	438.0
Wheat, ground	184.5	250.0
Wheat meal	30.0	25.0
Wheat middlings	28.0	–
Triticale, ground	14.0	–
Soybean meal (475 g CP kg${}^{-1}$)	280.9	173.0
Sunflower seed meal (385 g CP kg${}^{-1}$)	20.0	20.0
Rapeseed meal (357 g CP kg${}^{-1}$)	25.0	10.0
Corn DDGS (280 g CP kg${}^{-1}$)	15.0	30.0
Soybean oil	19.0	26.0
Limesterine	11.4	9.8
Monocalcium phosphate	10.7	7.4
Sodium chloride	2.42	2.37
Sodium bicarbonate	1.6	1.4
DL-methionine	0.85	1.05
L-Lysine	0.68	0.98
Threonine	0.35	–
Vitamin–mineral premix${}^{a}$	5.0	5.0
Chemical composition (g kg${}^{-1}$ of feed)		
DM (%)	90.63	90.01
CP	206.2	175.9
Crude fat	44.6	45.0
Crude fibre	42.0	43.4
Crude ash	50.5	47.3
N-free extracts	563.0	588.5
ME${}^{b}$ (MJ kg${}^{-1}$ of feed)	12.19	12.68
Calculated composition (g kg${}^{-1}$ of feed)		
Lysine	10.7	8.9
Methionine	3.9	4.1
Threonine	7.5	6.2
Tryptophan	2.6	2.1
Calcium	10.1	8.2
Phosphorus (soluble)	6.2	5.2

${}^{a}$ 1 kg of vitamin–mineral premix provided: 10 000 IU retinol, 2500 IU cholecalciferol, 20.00 mg α-tocopherol, 0.5 mg thiamine , 5.00 mg riboflavin, 20.00 mg niacinamide, 1.0 mg pyridoxine, 0.02 mg cobalamine, 0.5 mg folic acid, 2.5 mg menadione, 200.00 mg choline chloride, 45.00 mg Fe, 62.5 mg Mn, 50.00 mg Zn, 5.00 mg Cu, 0.25 mg Se, and 1.3 mg I. ${}^{b}$ The values are calculated from ingredient AME values. DM – dry matter, CP – crude protein, ME – metabolizable energy.

### 2.3 Evaluation of meat and liver quality

Female Muscovy ducks were slaughtered at 70 d of age, and Muscovy ducks (males) and mule ducks (males and females) at 84 d of age. A total of 40 birds were slaughtered for the tests (10 males and 10 females from each genetic group). Birds whose body weights were closest to the mean arithmetic body weight of males or females of a given genotype were selected for slaughter. Before slaughter, birds were fasted for 10 h but had unlimited access to water. Birds were slaughtered, defeathered and eviscerated on the farm. Birds were manually slaughtered by stunning with a club and cutting their blood vessels in the neck. The obtained carcasses were chilled for 18 h in a Hendi refrigerated cabinet (Hendi, Gądki, Poland) at 4 ${}^{\circ }$C. On removal from the refrigerator, each carcass was sampled for breast muscle (superficial pectoral muscle and deep pectoral muscle), leg muscle (all thigh and drumstick muscles), and liver to determine meat and liver quality traits.

The basic chemical composition (water, protein, fat, collagen content) of the breast and leg muscles of the ducks was determined by near-infrared transmission spectroscopy using calibration with artificial neural networks (ANN) with a FoodScan analyser (FoodScan Laboratory, Foss, Cheshire, UK).

The colour of meat was determined on the inner surface of raw breast muscles (superficial pectoral muscle) and leg muscles (thigh and drumstick muscles, following dissection of the patella and tendons and grinding) 24 h postmortem, after carcass chilling. The parameters $L^{*}$ – colour lightness and $a^{*}$ – relative redness, on a red–green axis, and $b^{*}$ – relative yellowness, on a yellow–blue axis were determined based on the CIE $L^{*}a^{*}b^{*}$ system (CIELab Color System, 1976). Colour variables were measured with a Konica Minolta CR-310 colorimeter (Konica Minolta, Japan). The measurement area was 50 mm in diameter, and the chroma meter was calibrated against a white reference tile (Y=92.80, x=0.3175, y=0.3333).

The amount of total pigments in the samples of breast and leg muscles was measured as haematin according to the procedure of Hornsey (1956). Filter optical density (E) was multiplied by 680 to calculate the concentration of meat pigments (haematin) in parts per million (ppm), i.e. micrograms of haematin per gram of meat.

Meat tenderness was determined by an Instron method using an Instron 3342 tensiometer (Instron Corp. USA) with a Warner-Bratzler (WB) tool. The test samples were stored frozen until determinations. After thawing, they were heated in a water bath to an internal temperature of 70 ${}^{\circ }$C. Heat treatment was done in a 0.85 % sodium chloride solution. Next, parallel to the orientation of the muscle fibres, cylindrical portions of muscle were cut using a cork borer, which were cut perpendicular to the orientation of the muscle fibres. The results were read as maximum shear force needed to shear the muscle fibres and expressed as newton (N) (Szalata et al., 1999). Each sample was determined four times (four replications), and the average was calculated for each sample. A total of 40 meat samples were evaluated, 10 from males and 10 from females of each genotype.

To determine the content of some minerals (sodium, potassium, magnesium, zinc, iron, copper), the meat and liver samples were lyophilized and wet mineralized in an Ethos Plus microwave digester (Milestone, Sorisole, Italy). The samples were analysed using an atomic absorption spectrometer (Thermo Scientific iCE 3000, Cambridge, United Kingdom). The meat samples were analysed for phosphorus content by the spectrophotometric method using a Marcel Media Eko spectrometer (Marcel, Warsaw, Poland). The mineral content in meat and liver were prepared and determined according to Polish standards.

Sensory properties were assessed on heat-treated meat obtained from the breasts. The meat samples were heat treated in 0.6 % brine solution to an internal temperature of 80 ${}^{\circ }$C, using a water to meat ratio of 2:1. After heat treatment, the samples were cooled to 60 ${}^{\circ }$C and evaluated (Krełowska-Kułas, 1993). The assessment was performed by a panel of six trained judges according to a scale provided by Baryłko-Pikielna and Matuszewska (2009). The following were assessed on a five-point scale: intensity of aroma and taste, with (1) point – imperceptible, (2) points – perceptible, (3) points – weakly distinct, (4) points – distinct, (5) points – very distinct; aroma and taste desirability, with (1) point – very undesirable, (2) points – undesirable, (3) points – neutral, (4) points – desirable, (5) points – very desirable; juiciness of meat, with (1) point – clearly dry, (2) points – slightly dry, (3) points – weakly juicy, (4) points – juicy, (5) points – very juicy; tenderness, with (1) point – very hard, (2) points – hard, (3) points – slightly tender, (4) points – tender, (5) points – very tender.

**Table 2:** Basic chemical composition of the breast and leg muscles of ducks of different genotypes.

Trait		Genotype (G) – sex (S)				SEM	P value		
		Muscovy		Mule			G	S	G × S
		Male	Female	Male	Female				
		(n=10)	(n=10)	(n=10)	(n=10)				
Water (%)	BM	72.2	72.0	71.3	70.9	0.2	<0.001	0.908	0.072
	LM	70.0	71.9	68.0	67.2	0.4	<0.001	0.070	0.002
Protein (%)	BM	25.0	24.7	26.3	27.2	0.3	0.051	0.537	0.083
	LM	23.1	21.4	22.1	23.0	0.2	0.055	0.024	<0.001
Fat (%)	BM	1.4	1.1	1.2	1.0	0.2	0.176	0.879	0.742
	LM	4.2	3.9	6.3	6.6	0.3	<0.001	0.977	0.332
Collagen (%)	BM	1.3	1.2	1.2	1.3	0.2	0.225	0.147	0.297
	LM	1.5	1.5	1.3	1.3	0.2	0.061	0.107	0.541

BM – breast muscle, LM – leg muscle.

**Table 3:** Content of some minerals in milligrams per 100 g of breast or leg meat from ducks of different genotypes.

Trait		Genotype (G) – sex (S)				SEM	P value		
		Muscovy		Mule			G	S	G × S
		Male	Female	Male	Female				
		(n=10)	(n=10)	(n=10)	(n=10)				
Na – sodium	BM	94.3	90.1	50.7	50.9	0.2	0.001	0.001	0.001
	LM	70.4	54.3	63.1	66.1	0.2	0.506	0.002	0.002
K – potassium	BM	359.4	352.2	321.4	344.6	0.2	0.455	0.142	0.032
	LM	330.0	331.2	276.1	286.2	0.4	0.944	0.002	0.002
P – phosphorus	BM	51.2	48.3	43.8	44.6	0.1	0.216	0.110	0.082
	LM	42.6	29.3	35.7	35.9	0.1	0.899	0.004	0.003
Mg – magnesium	BM	24.9	23.9	20.9	21.8	0.1	0.145	0.261	0.134
	LM	20.0	14.9	17.2	17.6	0.1	0.745	0.004	0.005
Zn – zinc	BM	0.7	0.5	0.8	1.2	3.6	0.109	0.828	0.806
	LM	2.1	2.5	3.0	3.0	4.3	0.004	0.327	0.316
Fe – iron	BM	5.8	5.2	5.6	5.3	7.6	0.929	0.637	0.898
	LM	2.5	2.5	2.6	2.6	2.2	0.265	0.869	0.870
Cu – copper	BM	0.3	0.3	0.3	0.4	0.4	0.234	0.483	0.340
	LM	0.1	0.3	0.3	0.3	0.4	0.590	0.005	0.006

BM – breast muscle, LM – leg muscle.

### 2.4 Statistical analysis

The numerical data collected for basic chemical composition, selected minerals, physicochemical and sensory traits of the meat, and mineral content in liver were subjected to statistical analysis. Arithmetic mean and standard error of the mean were calculated for each trait (together for both groups). Two-way analysis of variance was used to determine the effect of genotype and sex on the above meat characteristics of the ducks. To this end, the following linear model was used: $y_{ijk}=\mu +a_{i}+b_{j}+(a\times b{)}_{ij}+e_{ijk}$, where $y_{ijk}$ is value of the analysed trait, μ is overall means of the analysed trait, $a_{i}$ is effect of ith duck genotype, $b_{j}$ is effect of j – the sex, $(a\times b{)}_{ij}$ is the genotype by sex interaction, and $e_{ijk}$ is the random error.

The statistics for meat quality traits were calculated with SAS version 9.4 software (SAS Institute Inc., 2013). Significant differences between the compared ducks of different genotypes and between males and females were determined with Tukey's test. The level of significance was at P<0.05. The individual bird was the experimental unit for all analysed traits.

**Table 4:** Content of some minerals in milligrams per 100 g of liver from ducks of different genotypes.

Trait	Genotype (G) – sex (S)				SEM	P value		
	Muscovy		Mule			G	S	G × S
	Male	Female	Male	Female				
	(n=10)	(n=10)	(n=10)	(n=10)				
Na – sodium	103.0	85.5	75.5	56.2	0.2	0.026	0.002	0.823
K – potassium	220.0	203.0	219.1	195.4	0.2	0.544	0.008	0.626
P – phosphorus	57.5	46.8	56.3	43.4	0.1	0.291	<0.001	0.613
Mg – magnesium	19.8	14.5	15.4	12.9	0.1	0.079	0.029	0.356
Zn – zinc	4.4	4.0	4.7	3.7	1.0	0.284	<0.001	0.914
Fe – iron	57.5	46.8	47.1	54.5	8.0	0.001	0.001	0.173
Cu – copper	1.6	1.8	2.5	2.1	0.8	0.001	0.009	0.005

**Table 5:** Selected physicochemical traits of the breast and leg muscles of ducks of different genotypes.

Trait		Genotype (G) – sex (S)				SEM	P value		
		Muscovy		Mule			G	S	G × S
		Male	Female	Male	Female				
		(n=10)	(n=10)	(n=10)	(n=10)				
$L^{*}$ – lightness	BM	39.7	39.2	37.4	38.2	0.5	0.259	0.479	0.415
	LM	41.1	41.4	40.4	41.5	0.4	0.744	0.439	0.682
$a^{*}$ – redness	BM	16.5	17.3	15.8	15.2	0.4	0.046	0.678	0.068
	LM	15.3	16.8	15.8	15.0	0.4	0.362	0.675	0.117
$b^{*}$ – yellowness	BM	0.7	1.0	0.1	0.7	0.2	0.290	0.217	0.353
	LM	0.9	1.4	1.4	1.6	0.2	0.665	0.502	0.405
Haematin (µg g${}^{-1}$ meat)	BM	198.0	203.0	174.0	170.0	7.1	0.081	0.420	0.177
	LM	140.0	176.0	154.0	147.0	5.8	0.429	0.099	0.098
WB shear force (N)	BM	30.6	29.6	30.3	34.0	2.6	0.242	0.379	0.201
	LM	30.2	34.2	28.9	33.9	2.0	0.343	0.070	0.380

BM – breast muscle, LM – leg muscle.

## 3 Results and discussion

### 3.1 Chemical composition

Muscovy and mule ducks showed differences (P<0.05) in the water content of breast and leg muscles and in the fat content of leg muscles. Male and female Muscovy ducks had significantly more water in breast and leg muscles and less fat in leg muscles compared to mule ducks. Regardless of genotype, males exhibited significantly more protein in leg muscles. The genotype–sex interaction was significant for the water and protein content of leg muscles (Table 2). In a study by Wawro et al. (2004), Muscovy and mule ducks contained more water (75.5 %–77.1 %) and less protein (19.3 %–19.6 %), and the fat content of their breast muscles was similar to or lower (0.9 %–1.1 %) than the muscles of the ducks from our study. Another experiment (Khaziev et al., 2018) showed higher content of dry matter, protein, fat, and ash in breast muscles of mule compared to Muscovy ducks. In turn, Wołoszyn (2002) found more water and fat and less protein in the breast and leg muscles of 12-week-old mule males force-fed with steamed maize compared to the groups of ducks of different genotypes from our study. Omojola et al. (2014) found noticeably higher fat content (12.92 %), similar water content (71.64 %), and lower protein content (21.91 %) in breast muscles of Muscovy drakes compared to the breast meat of ducks from our study. Marzoni et al. (2014) reported similar protein content and higher water content in the muscles of Muscovy ducks compared to Muscovy ducks from our study. Larzul et al. (2006) observed similar water content and lower lipid and collagen content in the breast muscles of Muscovy, hinny, mule, and Pekin ducks compared to the amount of these components in breast meat of ducks from our study. The same authors reported significantly higher protein content in the breast muscles of 15-week-old Muscovy ducks compared to mule ducks. The analysis of the present results shows that breast muscles from mule ducks have a higher nutritive value than those from Muscovy ducks due to the higher protein and lower fat.

**Table 6:** Sensory properties of pectoralis major muscle in ducks of different genotypes.

Trait	Genotype (G) – sex (S)				SEM	P value		
	Muscovy		Mule			G	S	G × S
	Male	Female	Male	Female				
	(n=10)	(n=10)	(n=10)	(n=10)				
Aroma intensity (pts.)	4.4	4.3	4.4	4.1	0.1	0.314	0.572	0.576
Aroma desirability (pts.)	4.3	4.3	4.4	4.2	0.1	0.600	0.753	0.600
Juiciness (pts.)	3.7	3.6	3.8	3.7	0.1	0.702	0.700	0.930
Tenderness (pts.)	3.9	3.7	3.7	3.8	0.1	0.482	0.483	0.193
Taste intensity (pts.)	4.0	3.7	3.7	3.8	0.1	0.574	0.576	0.352
Taste desirability (pts.)	4.0	4.0	4.1	4.1	0.1	0.859	0.382	0.482

Duck genotype had a significant (P<0.05) effect on the sodium content of breast muscles and on the zinc content of leg muscles. Significantly higher sodium content was determined in the breast muscles of Muscovy ducks and zinc content in the leg muscles of mule ducks. Regardless of genotype, male breast muscles contained significantly more sodium than female breast muscles. The content of sodium, phosphorus, and magnesium in the leg muscles of males was significantly higher, and potassium, and copper content significantly lower than in female leg muscles. The genotype–sex interaction was significant for the sodium and potassium content of breast muscles and for the sodium, potassium, phosphorus, magnesium, and copper content of leg muscles (Table 3). In a study by Kokoszyński et al. (2017), breast muscles of Pekin ducks (SM3 Heavy hybrids from Cherry Valley Farms Ltd) aged 49 d contained more potassium and zinc and less iron compared to the analysed mule and Muscovy ducks. The same authors found a higher potassium, phosphorus, magnesium, and iron content in breast muscles compared to leg muscles, which is in agreement with our findings. The experiment of Lucia et al. (2008), as in our study, found lower zinc content in the meat of non-overfed Muscovy compared to mule ducks. Ismed et al. (2013) reported a significantly lower content of calcium, copper, sodium, and zinc in the meat obtained from whole carcasses of Muscovy ducks compared to the meat of Pekin and local Java ducks.

The compared genetic groups of the ducks differed (P<0.05) in the sodium, iron, and copper content of liver. Higher sodium and iron content and lower copper content were found in the liver of Muscovy compared to mule ducks. Regardless of genotype, males had significantly more sodium, potassium, phosphorus, magnesium, zinc, iron, and copper in the liver compared to females. The genotype–sex interaction was significant for the liver copper content (Table 4). Lucia et al. (2008) found higher zinc, copper, cadmium, and mercury content in the liver of non-overfed mule compared to Muscovy ducks. Duman et al. (2019) reported higher content (in terms of milligrams per 100 g liver) of sodium, potassium, phosphorus, iron, and zinc and similar or greater amount of copper in the liver of native Turkish ducks compared to the Muscovy and mule ducks under study. The same authors stated higher content of iron, copper, and calcium and lower content of potassium, magnesium, and zinc in the liver of ducks compared to the liver of chickens. Other studies (Lucia et al., 2008) showed the effect of origin, age, and diet of ducks on the mineral content of their livers.

### 3.2 Meat physicochemical properties

Analysis of the present results (Table 5) shows that the compared groups of the ducks differed significantly in the redness of breast muscles. Significantly higher redness values were found for the breast meat of Muscovy compared to mule ducks. The analysed groups of ducks did not differ significantly (P>0.05) in the values of colour lightness ($L^{*}$), yellowness ($b^{*}$), and WB shear force, in the haematin content of breast and leg muscles, and in the redness of leg muscles. The sex of birds and the genotype–sex interaction were not significant for the studied traits. Chartrin et al. (2006) found higher lightness ($L^{*}$) and yellowness ($b^{*}$) and lower redness ($a^{*}$) values for breast muscles of Muscovy compared to mule ducks. In our experiment, we found higher redness ($a^{*}$) and yellowness ($b^{*}$) values of breast muscles for Muscovy than mule ducks. The results could have been affected by the age of female Muscovy ducks, which were 2 weeks younger than the other birds under study. Fernandez et al. (2003) reported that selection of Muscovy duck for improved body weight and breast weight increased the lightness value of heavier breast muscle, which was associated with the dilution of haem pigments (myoglobin and haemoglobin) responsible for meat colour.

### 3.3 Meat sensory properties

Our study demonstrated a nonsignificant effect of genotype and sex on the sensory traits, i.e. aroma and taste intensity and desirability, and on the tenderness and juiciness of breast muscles (Table 6). Our study found nonsignificantly greater juiciness of breast muscles from male Muscovy and mule ducks with a higher fat content. The breast muscles of male Muscovy ducks with a higher fat content (P>0.05) were characterized by nonsignificantly greater tenderness and aroma intensity compared to female breast muscles. Nonsignificantly higher scores for aroma intensity, taste intensity, and tenderness of the breast muscles from Muscovy ducks may be indicative of their better (P>0.05) sensory quality compared to the breast muscles of mule ducks. Omojola (2007), which subjected the meat of ducks of different genotypes (Rouen, Pekin, Muscovy) to organoleptic evaluation, found the meat of Muscovy ducks to score lower for colour, aroma, tenderness, juiciness, and overall acceptability compared to the breast meat of Rouen ducks. Another experiment (Chartrin et al., 2006) demonstrated the effect of fat content on the sensory quality of the meat. Breast muscles of Muscovy ducks with the lowest fat content received the lowest scores for tenderness, juiciness, and aroma, which was not confirmed in our study. Conversely, Trembecká et al. (2017) reported the lowest scores for juiciness, tenderness, taste, and aroma of breast muscles from 42 d old Ross 308 broilers with the highest fat content. Baéza et al. (1998) stated lower juiciness and tenderness of breast meat from female compared to male Muscovy ducks, which is in agreement with our findings for this duck genotype.

## 4 Conclusions

In summary, mule ducks were characterized by significantly lower water and sodium content, redness of breast muscle, and also lower water and higher fat and zinc content in leg muscle, as well as higher copper and lower sodium and iron content in the liver compared to Muscovy ducks. The sex of birds had a significant effect on the mineral content (Na, K, P, Mg, Zn, Fe, Cu) of the liver and leg muscles (except for iron and zinc) and on the sodium content of the breast muscle.

## Data Availability

The data are available from the corresponding author upon request.
